# Epistatic Interaction of *CYP1A1* and *COMT* Polymorphisms in Cervical Cancer

**DOI:** 10.1155/2016/2769804

**Published:** 2015-12-21

**Authors:** Andreia Matos, Cindy Castelão, Alda Pereira da Silva, Irina Alho, Manuel Bicho, Rui Medeiros, Maria Clara Bicho

**Affiliations:** ^1^Genetics Laboratory and Environmental Health Institute, Faculdade de Medicina, Universidade de Lisboa, 1649-028 Lisbon, Portugal; ^2^Instituto de Investigação Científica Bento da Rocha Cabral, 1250-047 Lisbon, Portugal; ^3^Dermatology Research Unit, Instituto de Medicina Molecular, Faculdade de Medicina, Universidade de Lisboa, 1250-047 Lisbon, Portugal; ^4^Clinical and Translational Oncology Research Unit, Instituto de Medicina Molecular, Faculdade de Medicina, Universidade de Lisboa, 1250-047 Lisbon, Portugal; ^5^Molecular Oncology Group, Portuguese Institute of Oncology Porto Centre, 4200-072 Porto, Portugal

## Abstract

There is a clear association between the excessive and cumulative exposure to estrogens and the development of cancer in hormone-sensitive tissues, such as the cervix. We studied the association of* CYP1A1 M1* (*rs4646903*) and* COMT* (*rs4680*) polymorphisms in 130 cervical cancer cases (c-cancer) and 179 controls. The* CYP1A1* TT genotype was associated with a lower risk for c-cancer (OR = 0.39, *p* = 0.002). The allele C of* CYP1A1* was a risk for c-cancer (OR = 2.29, *p* = 0.002). Women with* COMT LL* genotype had a higher risk of developing c-cancer (OR = 4.83, *p* < 0.001). For the interaction of the* CYP1A1*&*COMT*, we observed that TC&HL genotypes had a greater risk for c-cancer (OR = 6.07, *p* = 0.006) and TT&HL genotypes had a protection effect (OR = 0.24, *p* < 0.001). The* CYP1A1 TT* and* COMT LL* genotypes had higher estradiol levels in c-cancer (*p* < 0.001 and *p* = 0.037, resp.). C-cancer is associated with less production of 2-methoxy-estradiol resultant of functional polymorphisms of* CYP1A1* and* COMT*, separately.* CYP1A1* and* COMT* work in a metabolic sequence and their interaction could lead to an alternative pathway of estrogen metabolism with production of 16-OH-estrone that is more proliferative.

## 1. Introduction

Cervical cancer is the fourth most common cause of cancer in women worldwide [1]. The association between human papillomavirus (HPV) infection and the development of cervical cancer has been established [2]. These viruses have a sexual transmission and can cause cancer in the cervix, anus, vulva, vagina, penis, and oropharyngeal cancers [3, 4]. Cervical cancer arises via a series of four steps: HPV transmission, viral persistence, progression of a clone of persistently infected cells to precancer, and invasion [5]. Backward steps may occur, namely, clearance of HPV infection. Regression of precancer to normality may also occur but is less frequent [5]. Cervical cancer develops slowly; it is often established over a decade after initial infection with high-risk HPV and only arises in those women whose infections do not resolve spontaneously. Infection by high-risk HPV is necessary but not sufficient for progression to cancer [6]. Mutations in cellular genes and chromosomal rearrangements induced by genomic instabilities are important contributing events [2, 3, 7]. There is a clear association between the excessive and cumulative exposure to estrogens and the development of cancer in hormone-sensitive tissues [7]. The carcinogenesis associated with estrogens may be the result of multiple overlapping mechanisms. One major pathway is the extensively studied hormonal pathway, by which estrogen stimulates cell proliferation through nuclear estrogen receptor-mediated signaling, thus resulting in an increased risk of genomic mutations during DNA replication. However, estrogen can also lead to cancer through its metabolism, producing reactive species of oxygen (ROS) and metabolites that form DNA adducts [8–10]. Two pathways can metabolize estrogen: the 16*α*-hidroxyestrone pathway and the formation of catechol-estrogens [11]. The pathway that leads to the formation of catechol-estrogens starts with the hydroxylation of estrogens, reaction mediated by the enzymes CYP1A1 and CYP1B1, which yields 2-hydroxy-estrogen (2-OH-CE) and 4-hydroxy-estrogen (4-OH-CE), respectively [8, 12]. Catechol-estrogens may suffer oxidation, forming semiquinones and quinones, in a process that leads to the formation of ROS and DNA adducts, that may damage the DNA, resulting in mutations [8, 10, 13, 14].

However, catechols can be methylated by COMT resulting in metabolites that do not yield quinones or ROS. In fact, the methylation of 2-hydroxy-estrogen leads to the formation of 2-methoxy-estrogen, which has been shown to have antiapoptotic and antiangiogenic properties [10]. Estrogen receptors may also influence cervical cancer by binding to an ERE (estrogen responsive element) in the viral genome, leading to an increased expression of oncogenic HPV genes of E6 and E7 [15, 16]. Therefore, CYP1A1 and COMT are important enzymes in the metabolism of estrogen. The role of CYP1A1 as a key enzyme involved in the metabolic activation of polycyclic aromatic hydrocarbons is very well established. The gene for this enzyme is located in the long arm of chromosome 15 (15q22–q24) [17, 18]. The polymorphism in study, designated M1, gives rise to an Msp1 restriction site in the 3′ noncoding region at T6235C and has been reported to significantly elevate the enzymatic activity compared with the wild type genotype [14, 17].* COMT* is an ubiquitous enzyme; it uses a methyl group from* SAM* (S-adenosylmethionine) to methylate a great variety of compounds, including catecholamines. Its gene is located in the long arm of chromosome 22 (22q11.2) and it presents a functional polymorphism G-A in codon 158, resulting in a change of amino acids in the protein, valine to methionine. This functional polymorphism contributes to 3- to 4-fold decrease in methylation activity of the COMT enzyme and distinguishes the COMT-H (high activity) and COMT-L (low activity) alleles. Thus, with regard to the predicted enzymatic activity of the protein, genotypes are designated as high (COMT-HH), intermediate (COMT-HL), and low (COMT-LL) [19–21].

So, an association study of functional polymorphisms involved in synthesis of 2-methoxy-estradiol, namely,* CYP1A1* (*rs4646903*) and* COMT* (*rs4680*), could contribute to understanding the role of estrogen in cervical cancer.

## 2. Materials and Methods

### 2.1. Subjects

This investigation was conducted as part of a case-control study of genetic polymorphisms in cervical cancer. Written informed consent was obtained from all participants. The 130 participating women had cervical cancer with ages ranging from 19 to 77 years (mean: 42.75 ± 13.18 years) and only 35.1% had smoking habits.

At the first visit, women in the study group were observed after evaluation of diagnostic tests, in order to make the correct clinical stage cancer (Protocol Gynecology Oncology Service). Women referenced by precancerous lesions were examined by colposcopy for identification of these lesions and then the cytology/histology was performed. All original histological examinations (preneoplastic lesions and cancer) were consigned by the Pathology Service of Virology Laboratory of the Portuguese Institute of Oncology.

Control individuals were selected as part of an ongoing project of effects of exercise on cardiovascular risk marker on healthy women, namely, sports as water aerobics and step. The 179 healthy women recruited had mean of 60.75 ± 6.34 years (ages ranging from 22 to 79) and only 5.2% had smoking habits.

### 2.2. Genomic DNA Isolation

Whole blood samples from patients and controls were stored with EDTA at −20°C. The genomic DNA was isolated through a nonenzymatic method adapted from Lahiri and Numberger (1991) method [22].

### 2.3. Genotyping of* CYP1A1 M1* (*rs4646903*)

The* CY1A1* genotypes were determined by the polymerase chain reaction (PCR) technique; the polymorphic region was amplified in a 50 *μ*L reaction mixture: 10 mM of each primer (forward: 5′-CCTTCTTGCTGGCACCCCAT-3′ and reverse: 5′- GGAAGTCCAAAACTCGCACCA-3′), 200 ng of genomic DNA, and 0,2 mM of PCR* nucleotide Mix* Thermo Scientific* DreamTaq Green* containing 10 mM dNTPs, 1,5 mM MgCl_2_, and 1 U* Taq* polymerase. PCR conditions involved an initial denaturation of DNA at 94°C for 2 min (*hot start*), followed by 35 cycles of amplification at 94°C for 30 sec (melting), 58°C for 30 sec (annealing), and 72°C (extension) for 75 sec. The amplified fragments of 899 bp were then digested by the restriction endonuclease Msp1 at 37°C for 16 h according to the manufacturer's recommendations. The digestion products were analyzed by electrophoresis in 3% agarose gel stained with ethidium bromide (10 *μ*g/mL) for 60 minutes, with 80 volts. With this process we are able to differentiate genotypes: the TT genotype gives rise to one single band of 899 bp; the CC genotype appears as two bands, one with 693 bp and the other with 206 bp; the TC genotype has all three bands.

### 2.4. Genotyping of* COMT* (*rs4680*)

Using the PCR technique, the polymorphic region was amplified in a 50 *μ*L reaction mixture: 10 mM of each primer (forward: 5′-GGCTCATCACCATCGAGATCAA-3′ and reverse: 5′-CCAGGTCTTGACAACGGGTCA-3′), 200 ng of genomic DNA, and 0,2 mM of PCR* nucleotide Mix* Thermo Scientific* DreamTaq Green* containing 10 mM dNTPs, 1,5 mM MgCl_2_, and 1 U* Taq* polymerase. PCR conditions involved an initial denaturation of DNA at 94°C for 2 min (*hot start*), followed by 35 cycles of amplification at 94°C for 45 sec (melting), 60°C for 45 sec (annealing), and 72°C (extension) for 60 sec. After the 35 cycles, there is a final extension phase for 7 minutes at 72°C. The amplified fragments were then digested by the restriction endonuclease NlaIII at 37°C for 18 hours and 20 min at 65°C according to the manufacturer's recommendations. The digestion products were analyzed by electrophoresis in 3% agarose gel stained with ethidium bromide (10 *μ*g/mL) for 90 minutes, with 85 volts. With this process we are able to differentiate genotypes: the HH genotype gives rise to two bands, one of 111 bp and the other of 89 bp; the LL genotype appears as one single band, with 71 bp; the HL genotype has all three bands.

### 2.5. Estradiol

The levels of estradiol were determined by ELISA kit (R&D Systems) according to manufacturer's instructions.

### 2.6. Statistical Analysis

Observed genotype frequencies were tested for deviation from Hardy-Weinberg equilibrium (HWE) with the Chi-square goodness-of-fit test (*χ* ^2^). This test was also used to evaluate the significant differences between the two populations, in order to know if the odds ratio (OR) test was justifiable. OR for cervical cancer risk and the corresponding 95% confidence intervals (95% CI) were calculated using logistic regression analysis. This test was applied to each polymorphism, to analyze its risk factor individually; it was also applied to the association of both polymorphisms to analyze the epistatic relation between these enzymes and how it may affect the risk for cervical cancer. For pairwise comparisons between groups we used Mann-Whitney *U* test. All statistical analyses were carried out using the SPSS 20.0 software. *p* < 0.05 was considered statistically significant.

## 3. Results

We performed the determination of* CYP1A1* (*rs4646903*) and* COMT* (*rs4680*) polymorphisms in 130 women with cervical cancer and 180 healthy women. The control group had significantly higher ages than patients (*p* = 0.001), being the majority with age higher than 40 years old (*p* = 0.029). Actually, most of women from control group were postmenopausal (≥50 years old) (69.4%), contrarily to cervical cancer women (age < 49 years old, 72.8%) (*p* < 0.001) (data not shown).

For estradiol levels, we observed that women with cervical cancer had higher levels (Table 3), *p* < 0.001.

### 3.1. *CYP1A1 M1* (*rs4646903*)

Allele and genotype frequencies in cervical cancer and healthy women for* CYP1A1 M1* polymorphism are shown in Table 1. The genotype distributions in control group were in HWE equilibrium (*p* > 0.05). We observed significant differences between cervical cancer patients and healthy women (*p* = 0.006). The* CYP1A1 TT* genotype was more frequent in control group (79.8%) (Table 1). On one hand, individuals carrying the TC genotype were 2.29-fold at a higher risk for developing cervical cancer compared with those having homozygous association of* CYP1A1 CC*&*TT* genotypes (OR = 2.29, 95% CI = 1.25–4.20, *p* = 0.007). On the other hand, the association of* CYP1A1 TC*&*CC* genotypes represents almost 3-fold risk for patients (OR = 2.54, 95% CI = 1.41–4.57, *p* = 0.002) (data not shown). Indeed, we observed significant differences in distribution of* CYP1A1* allelic frequencies (*p* = 0.002), where the allele C is a risk for cervical cancer (Table 1). However, all these results lose statistical significance when adjusted for age (*p* > 0.05) (Table 1).

We also evaluate the levels of estradiol in relation to the studied polymorphisms (Table 3). According with the following model as* CYP1A1 TC*&*CC* and* CYP1A1 TT* genotypes separately, we observed significant higher values of estradiol in cervical cancer (*p* > 0.001) (Table 3). Although not statistically significant, the values of estradiol in patients were slightly higher in* CYP1A1 TT* genotype (data not shown) (Table 3).

### 3.2. *COMT* (*rs4680*)

Table 2 represents the distribution of* COMT* genotypes and allelic frequencies between cervical cancer patients and healthy women. Using *χ* ^2^ test, we established that the control group was in HWE (*p* > 0.05). We observed statistically significant differences in genotype and allelic frequencies (*p* = 0.003 and *p* = 0.015, resp.). The* COMT HH* and* HL* genotypes were more frequent in control group and* COMT LL* genotype in patients (Table 2). The allele H was more frequent in controls contrary to cervical cancer patients (*p* = 0.015, 70%* and* 40%, resp.). Indeed, when adjusted for age, the allele H was protector for cervical cancer (OR = 0.34, 95% CI = 0.21–0.56, *p* < 0.001), where the allele L is a risk, which could represent up to 3 times more risk (OR = 2.93, 95% CI = 1.80–4.78, *p* < 0.001).

For adjusted OR, the* COMT HH* genotype seems to be protector for cervical cancer (OR = 0.31, 95% CI = 0.15–0.65, *p* = 0.002), but the* COMT* polymorphism protection role was more statistically significant when associated the* COMT HH*&*HL* genotypes (OR = 0.21, 95% CI = 0.09–0.48, *p* < 0.001) (data not shown). When evaluating the risk for cervical cancer associated with the* COMT* genotypes, we observed that the best model was the* COMT LL* genotype, which could represent up to 5 times more risk (OR = 4.83, 95% CI = 2.08–11.20, *p* < 0.001).

For the levels of free estradiol, we found higher values for patients compared to healthy women, when considering the* COMT HH*&*HL* and* COMT LL* genotypes, separately (Table 3). Although not statistically significant, we observed that cervical cancer patients had higher values of estradiol in* COMT LL* genotype (data not shown) (Table 3). When adjusted for estradiol's levels, we did not find statistically significant differences in* COMT* polymorphism in our sample (data not shown).

### 3.3. Interaction of* CYP1A1* (*rs4646903*) and* COMT* (*rs4680*) Polymorphisms in an Epistatic Relation

To investigate whether profiles of* CYP1A1* and* COMT* genotypes might be associated with the risk of cervical cancer, we examined the effect combinations of genotypes.

Because the enzymes* COMT* and* CYP1A1* are closely related and work in metabolic sequence in the metabolism of estrogens, it is important to examine if the functional polymorphisms of these enzymes in association change the risk to develop cervical cancer in the carriers. In Table 4 it is shown the different associations of* CYP1A1*&*COMT* genotypes between patients and healthy women. From all possible associations, we identified two models that best fit as protection or risk in cervical cancer. The highest risk for cervical cancer was for the association of* CYP1A1*&*COMT TC*&*HL* (OR = 6.07, 95% CI = 1.67–22.09, *p* = 0.006), without adjusting for age. We also observed that the* CYP1A1*&*COMT TC*&*HH* might increase the risk for cervical cancer, though with a wide confidence interval (OR = 13.67, 95% CI = 1.72–109.39, *p* = 0.014) (data not shown). In the other side, for protection we identified the* CYP1A1*&*COMT TT*&*HL* as a best model, even when adjusted for age (OR = 0.18, 95% CI = 0.06–0.53, *p* = 0.002).

Finally, when we adjusted these models for estradiol levels, we observed that the* CYP1A1*&*COMT TT*&*HL* genotypes maintain their protection role for cervical (OR = 1.32; 95% CI = 1.12–1.55, *p* = 0.001).

## 4. Discussion

This is the first study in Portugal that exposes the interaction of CYP1A1 and COMT polymorphisms in cervical cancer.

In the current study, it was shown that women with cervical cancer have the genotypes of* CYP1A1 M1* TC&CC, which represented almost 3-fold risk when associated to* CYP1A1* CC genotype, in a dominant model (Table 1). Indeed, the allele C of* CYP1A1* features associated to risk for cervical cancer, which contributes to a higher activity expression of the enzyme. So, according to other authors, the carriers of these genotypes may have a higher concentration of catechol-estrogens, when compared to the genotype TT that represents a lower inducible activity enzyme [18, 20].

Also in relation to the metabolism of estrogens, the* COMT* appears to be an important enzyme on metabolisms of phase two (II) [23]. In our study, the allele L of* COMT* G158A gives rise to a lesser enzymatic activity, being a risk factor, since it is associated with a higher production of 4-OH-CE, a catechol-estrogen, that may enter redox cycles to form quinones that may lead to DNA lesions. The catechol oxidation to quinones is a competitive reaction with its methylation; the diminished activity of COMT will lead to a greater risk of mutation by ROS lesion or DNA adducts [19]. The low activity of* COMT*, associated with the mutant allele L, was linked to both benign and malignant disease, such as endometriosis, endometrial cancer, and breast cancer [19]. This low activity is also associated with less production of 2-methoxyestradiol, an endogenous inhibitor of hypoxia inducible factor 1*α* and microtubules [24, 25].

Since the* CYP1A1* and* COMT* work in a metabolic sequence, we evaluated their epistatic interaction. Table 4 showed that the association of the* CYP1A1*&*COMT* TC&HL genotypes leads to a higher risk for cervical cancer. The result of this association is, therefore, completely different from the result obtained when the polymorphisms were studied individually, namely, for* COMT* polymorphism. For protection model, we identified the* CYP1A1*&*COMT* TT&HL genotypes as a possible model that best fit on protection for cervical cancer (Table 4). In both models,* CYP1A1*&*COMT* TT&HL for risk or protection effects on cervical cancer were dependent on estradiol levels.

This variability in the genetic associations suggests that, besides the genetic basis, there are more factors that contribute to the COMT enzyme activity. Many studies have established that the hypermethylation of CpG islands in* COMT*'s promoter is linked to hypoexpression of the gene in some cancers. Actually, Sasaki et al. observed that the promoter's methylation in the COMT gene was superior in tumoral tissue than normal tissue [12, 26]. COMT methylates 2-OH-CE, product of CYP1A1, faster than 4-OH-CE, product of CYP1B1, leaving the latter free to enter redox cycles and to turn into quinones. 4-OH-CE quinones have a bigger half-life than 2-OH-CE quinones, and 4-OH-CE has been shown to have more estrogenic and mutagenic effects than 2-OH-CE [14, 20]. The result obtained may also be due to the negative feedback that the methylated products of COMT have on CYP1A1's activity and CYP1B1's activity. This inhibition probably leads to a shift in the pathways of detoxification of estrogens to the 16*α*-hydroxylation pathway, catalyzed by CYP3A4. This pathway presents proliferative effects on cells, which could contribute to an increase in the risk for cervical cancer [26, 27].

In our study, we also observed that the* CYP1A1* polymorphism seems to be somehow affected by age, contrarily to what was observed for* COMT* polymorphism, due the more dependency on hormone induction of CYP1A1 by aryl hydrocarbon receptor (AhR) [28]. Thus, the increased susceptibility induced by the studied polymorphism for cervical cancer might be associated with a greater immune depression associated with AhR [29].

Knowing that estradiol stimulates the growth of HPV-positive cervical cancer cells [15] and as described by other authors, the levels of free estradiol are higher when the activity of* COMT* were low (COMT LL genotype) [30], contrary to* CYP1A1.* Noteworthy, most of cervical cancer women were perimenopausal, which is consistent with higher levels of estradiol, ultimately, within COMT polymorphism, affecting the formation of 2-methoxy-estradiol and immune system [31].

Actually these results may have a plausible explanation since these enzymes work in metabolic sequence in the metabolism of estrogens.

## 5. Conclusion

The study of the COMT and CYP1A1 polymorphisms individually resulted in predictable results according to the published works. The lower activity of* COMT* represents a higher risk for cervical cancer and so does the highest activity of* CYP1A1*. However, when we studied the interaction of these polymorphisms, our results were apparently contradictory. We observed that the genotypes for* CYP1A1* and* COMT* for intermediate activity represented the highest risk for developing cervical cancer of that observed individually for COMT genotype that was protector. The* COMT* polymorphism possibly represents a more important role for protection than that of risk for cervical cancer. The other possible model for risk, namely, the* CYP1A1*&*COMT* TC&HH genotypes, may be due to a series of facts: either the methylation of COMT's promoter that causes hypoexpression of the enzyme or the fact that COMT has a highest affinity for 2-OH-CE, leaving 4-OH-CE free to enter redox cycles and to suffer oxidation to more mutagenic quinones. It can also be due to the negative feedback that methylated products of COMT have on CYP1A1 and CYP1B1; this inhibition probably leads to a shift in the estrogen metabolizing pathways, causing proliferative effects on cells, which could contribute to an increase in the risk for cervical cancer. Therefore, there are still studies to be done in this area to truly understand the role of estrogens and key enzymes in its metabolism in cervical cancer.

These results can help to define an individualized tumor prevention and therapy based in characteristics of the host and to contribute to the understanding of the oncogenesis behind cervical cancer.

## Figures and Tables

**Table 1:** Distributions of *CYP1A1 M1* (*rs4646903*) polymorphism in cervical cancer cases and controls.

*CYP1A*1 *rs4646903*	OR [95% CI]						
	Controls	Cervical cancer	*p*	OR crude^r^	OR adjusted^a^	*p* ^r^	*p* ^a^
	*n* = 124	*n* = 105					
*TT*	99 (79.8)	64 (61.0)	**0.006**	0.39 [0.22–0.71]	0.66 [0.27–1.66]	**0.002**	0.381
*TC*	23 (18.5)	36 (34.3)		2.29 [1.25–4.20]	1.25 [0.48–3.27]	**0.007**	0.655
*CC*	2 (1.6)	5 (4.8)		3.05 [0.58–16.06]	3.16 [0.33–30.22]	0.188	0.317

*T*	221 (89.0)	164 (78.0)	**0.002**	0.44 [0.26–0.73]	0.63 [0.29–1.39]	**0.002**	0.255
*C*	27 (11.0)	46 (22.0)		2.29 [1.37–3.85]	1.58 [0.72–3.48]	**0.002**	0.255

The values for the genotypes and respective allele frequencies represent absolute frequencies (relative frequencies, %). *p*: *χ* ^2^ test values; OR: odds ratio; *p* ^r^: values for OR crude; *p* ^a^: values adjusted for age (regression binary logistic); values statistically significant for *p* value < 0.05.

**Table 2:** Distribution of *COMT G158A* (*rs4680*) polymorphism in cervical cancer cases and controls.

*COMT rs4680*	OR [95% CI]						
	Controls	Cervical cancer	*p*	OR crude^r^	OR adjusted^a^	*p* ^r^	*p* ^a^
	*n* = 157	*n* = 108					
*HH*	79 (50.3)	48 (44.4)	**0.003**	0.79 [0.48–1.29]	0.31 [0.15–0.65]	0.347	**0.002**
*HL*	63 (40.1)	34 (31.5)		0.69 [0.41–1.14]	1.10 [0.55–2.19]	0.152	0.798
*LL*	15 (9.6)	26 (24.1)		3.00 [1.50–5.99]	4.83 [2.08–11.20]	**0.002**	**<0.001**

*H*	221 (70.0)	130 (60.0)	**0.015**	0.64 [0.44–0.92]	0.34 [0.21–0.56]	**0.015**	**<0.001**
*L*	93 (30.0)	86 (40.0)		1.57 [1.09–2.26]	2.93 [1.80–4.78]	**0.015**	**<0.001**

The values for the genotypes and respective allele frequencies represent absolute frequencies (relative frequencies, %). *p*: *χ* ^2^ test values; OR: odds ratio; *p* ^r^: values for OR crude; *p* ^a^: values adjusted for age (regression binary logistic); values statistically significant for *p* value < 0.05.

**Table 3:** Comparison of estradiol levels between controls and cervical cancer cases for different models of *CYP1A1* (*rs4646904*) and *COMT* (*rs4680*) polymorphisms.

*CYP1A1*&*COMT* *rs4646903/rs4680*	Controls	Cervical cancer	*p*
Estradiol (pg/mL)	95 (7.17 ± 0.77) [0.95–31.64]	28 (48.71 ± 12.35) [1.19–212.39]	**<0.001**

*CYP1A1 TT*	70 (6.80 ± 0.89) [0.95–31.64]	16 (50.66 ± 17.56) [1.19–212.39]	**<0.001**
*CYP1A1 TC*&*CC*	15 (7.62 ± 1.53) [1.87–17.90]	12 (46.14 ± 17.57) [19.53–189.76]	**<0.001**

*COMT HH*&*HL*	73 (8.46 ± 6.39) [0.95–412.22]	19 (47.66 ± 33.11) [1.19–641.15]	**<0.001**
*COMT LL*	11 (8.19 ± 27.32) [2.43–272.39]	4 (99.09 ± 176.48) [21.00–764.57]	**0.037**

The values represent *N* (median ± standard error) [min.–max.].

*p*: Mann-Whitney test; values statistically significant for *p* value < 0.05.

**Table 4:** Represents the cumulative effects of association of *CYP1A1 M1* and *COMT* polymorphisms between cervical cancer cases and controls.

*CYP1A1*&*COMT rs4646903/rs4680*	OR [95% CI]					
	Controls	Cervical cancer	OR crude	OR adjusted^a^	*p* ^r^	*p* ^a^
Other genotypes^b^	45 (44.6)	64 (77.1)	4.19 [2.20–7.99]	5.68 [1.88–17.13]	**<0.001**	**0.002**
Protection model *TT*&*HL*	56 (55.4)	19 (22.9)	0.24 [0.13–0.46]	0.18 [0.06–0.53]		

Other genotypes^c^	98 (97.0)	70 (84.3)	0.17 [0.05–0.60]	0.13 [0.01–1.31]	**0.006**	0.083
Risk model *TC*&*HL*	3 (3.0)	13 (15.7)	6.07 [1.67–22.09]	7.65 [0.76–76.49]		

The values for the genotypes represent absolute frequencies (relative frequencies, %). OR: odds ratio; *p* ^r^: values for OR crude; *p* ^a^: values adjusted for age (regression binary logistic); values statistically significant for *p* value < 0.05.

In protection model, the other genotypes represent (b) *TT*&*LL*, *TC*&*LL*, *CC*&*HL*, *TT*&*HH*, *TC*&*HL, TC*&*HH*; *CC*&*HH, and CC*&*LL*.

In risk model, the other genotypes represent (c) *TT*&*HL*, *TT*&*LL*, *TC*&*LL*, *CC*&*HL*, *TT*&*HH*, *TC*&*HH*, *CC*&*HH, and CC*&*LL*.
